# Universal Design: A Step toward Successful Aging

**DOI:** 10.1155/2013/324624

**Published:** 2013-01-30

**Authors:** Kelly Carr, Patricia L. Weir, Dory Azar, Nadia R. Azar

**Affiliations:** ^1^Department of Kinesiology, University of Windsor, 401 Sunset Avenue, Windsor, ON, Canada N9B 3P4; ^2^Dory Azar Architect, 870 Revland Drive, Tecumseh, ON, Canada N8N 5B2

## Abstract

The concept of aging successfully has become increasingly important as demographics shift towards an aging population. Successful aging has been defined to include (1) a low probability of disease and disease-related disability; (2) a high level of physical and cognitive functioning; and (3) an active engagement in life. The built environment can create opportunities or constraints for seniors to participate in social and productive activities. Universally designed spaces are more easily accessed and used by a spectrum of people without specialized adaptations. Thus, a universally designed environment creates opportunities for older adults to participate in these activities without the stigmatization associated with adapted or accessible designs. Providing older adults with specific universal design options (e.g., lever handle faucets) has the potential to increase the ease of completing activities of daily living, which promotes a continual engagement in life. Literature regarding universal design is promising; however, its theory requires further attention from professionals designing the built environment, evidence of the significance of its application from academics, and the embracement of its core principles from society. Overall, universal design has the potential to provide a stepping stone toward successful aging.

## 1. Introduction

A rise in life expectancy and a decline in fertility rates have created a shift in demographics leading to an aging population [1–4]. Currently, Canada's population of citizens 65 years of age and older is at a record high (14.8%; [3]). This older adult population has experienced a 14.1% growth in the past five years, with 60–64 year olds experiencing the greatest increase, followed by centenarians [3]. If this trend continues, seniors will account for nearly a quarter of the population by 2036 [4]. At that point, the number of older adults will surpass the number of children, a first in Canadian history [4]. These demographic trends span beyond Canadian borders and have been recognized globally [1]. As a result, successful aging has become an important concept worldwide [5]. 

Successful aging has been empirically defined to include (1) a low probability of disease and disease-related disability; (2) a high level of physical and cognitive functioning; and (3) an active engagement in life [6, 7]. To some extent, these components represent a hierarchical relationship, as it is suggested that the absence of disease and disability leads to a prolonged maintenance of physical and cognitive functioning, which enables a higher level of engagement defined as the combination of social activity and productive activity [7, 8], participation in leisure activities [9], and belonging to neighborhood groups [10] (see [11] for an extensive review). Individuals who meet these hierarchical components during the aging process maintain the capacity to adequately function during daily living, leading to greater independence [12]. Continued independence is suggested to be an important factor throughout the aging process as it facilitates control and autonomy, both of which increase well-being and life satisfaction [6, 13]. In addition to the psychosocial benefits associated with successful aging, the absence of chronic disease seen in older adults who have aged successfully has the potential to reduce the health care costs required for an aging population [14]. While there are older adults who are successfully aging, the majority of older adults are dealing with some kind of limitation. Putting these ideas into a practical setting, we need to consider older adults, living in the broader community who are active in their homes, church groups, recreation centers and are out and about in their community doing errands to manage their personal lives (e.g., shopping, banking, appointments, etc.). All of these represent forms of engagement. Though planning and design of the built environment has long been considered an influential factor on the aging process, only recently has the philosophy of universal design flourished [15, 16]. Universal design includes designing environments and products that are more easily accessed and used by a spectrum of people without specialized adaptations [16]. A key concept of universal design is to provide accessibility without stigmatization, by integrating accessibility features such that they benefit all users while going essentially unnoticed [16]. For example, automatic sliding doors are standard features at many shopping centers. These doors remove barriers to entry for individuals in a wheelchair, and for older adults or young children who have difficultly opening heavy doors. However, they also benefit all customers carrying purchased goods and are a convenience feature for the able-bodied individual with free hands. They provide accessibility without stigmatization, since everyone entering or exiting the store uses them, regardless of ability. 

The concept of universal design proves to be timely, as it complements the implementation of the Accessibility for Ontarians with Disabilities Act (2005), which requires full accessibility of public and private buildings by the year 2025 [17]. While being mindful of complete accessibility, universal design aims to accommodate human diversity with a focus on inclusion and equality [18]. It is these aspects of universal design that set it apart from accessibility design and specialized adaptation and create the potential to further enhance well-being and quality of life [19]. Specific to older adults, implementing universal design could enable individuals to continue to complete activities of daily living independently while providing a safer environment for daily functioning and navigation, both of which can reduce the chances of an older adult entering a nursing home [19, 20]. These benefits are provided through the implementation of any of the seven structured universal design principles outlined in Table 1 [16]. For example, providing automatic sliding doors at the entrance of a public building meets at least 6 of these principles. As described in the previous paragraph, they provide equal access for individuals with diverse abilities and needs (Principles 1 and 2). They are very simple and intuitive to use (Principle 3), and since they open when sensors detect the presence of a person within range (as opposed to having to open the door manually or even by pushing a button), they require no physical effort to operate (Principle 6). These sensors also ensure that the sliding door will remain open for as long as the person remains within the sensor's range, minimizing the risk of injury due to the door shutting before they are able to walk all the way through (Principle 5). Finally, automatic sliding doors are frequently much wider than a typical swinging door, making it easier to maneuver through the door while using ambulatory aids (e.g., canes, walkers, scooters, wheelchairs, crutches, etc.) or while manipulating other large items (e.g., carrying large/irregular packages, pushing a stroller, or shopping cart) (Principle 7). For older adults who no longer have enough upper limb strength to open a heavy door, and/or who use a walker or a wheelchair, and/or may have age-related visual impairments, and/or move slowly; automatic sliding doors remove a significant barrier to entering public buildings independently and facilitate their ability to engage in the activities within. 

 As the population experiences a substantial increase in older adults it is imperative to understand the impact thoughtful design and planning has on the ability to age successfully [3, 4]. Specifically, universal design may have the potential to affect older adult's engagement with life, by providing accessible and accommodating environments in which this population can thrive without stigmatization. As Rowe and Kahn [6] proposed, it is the environment surrounding the older adults (i.e., residential and community environment) that is the primary factor that creates opportunities and constraints for the individual. Thus, modifications to the built environment can impact the engagement levels (i.e., interpersonal relations and involvement in productive activities) of older adults to promote successful aging [7]. This approach is favorable, as it increases an individual's functional abilities with the least amount of effort required from the individual when compared to approaches attempting to change the person (i.e., exercise to increase strength, to enable the individual to operate a regular door) or provide assistive tools (i.e., provide an electronic push button to open the door) [16]. Therefore, the aim of this review is to conceptualize the importance of older adults actively engaging in life, the roles the built environment and universal design play in maintaining engagement, and provide examples to implement this approach for optimal engagement in late adulthood. Through this review, we hope to bring attention to the potential for universal design to promote successful aging by facilitating active engagement in one's life. 

## 2. Active Engagement in Life

### 2.1. Predictor of Successful Aging

While Rowe and Kahn's [7] definition of successful aging has been the most widely empirically tested, their description of active engagement is limited to the maintenance of interpersonal relationships and continued involvement in productive activities. Interpersonal relationships are connections and interactions with others, including the availability of emotional support and physical assistance, whereas productive activities are all activities that provide value regardless of compensation to the individual (i.e., volunteering, paid employment; [7]). Though Rowe and Kahn's definition of engagement in life is highly regarded in academia, it should be noted that there is little consensus regarding this concept [21]. As highlighted by Levasseur et al. [11], it might be thought of as a continuum ranging from social participation to social engagement, with activities being performed by oneself, with others, or for others. 

Despite this inconsistency, continued engagement is commonly regarded as a significant predictor of successful aging [21]. Specifically, it is suggested that older adults perceive social and familial relationships as fundamental to aging successfully [22–24]. Older adults residing in nursing homes also emphasize the importance of social interactions [25]. This view may be a product of the companionship and sustained connection to the broader community these contacts offer [26]. Complementing interpersonal relations with involvement in productive activities has also been perceived as beneficial; a sample of older adults reported participating in activities (i.e., gardening) to be among the most important factors required to age successfully [27]. Therefore, continued engagement in life is not only valued within empirical definitions of successful aging, it is perceived by older adults to be pertinent to the aging process as well. 

### 2.2. Benefits of Engagement

Engagement in life has received the least research attention of the three components of successful aging described by Rowe and Kahn [7]; however, the benefits obtained through continued engagement have not gone unnoticed [21, 28]. Research suggests that a positive correlation exists between the number of activities older adults participate in and quality of life and well-being [29, 30]. This relationship is even more pronounced for individuals with minimal contact with family members, individuals suffering from functional loss, and during bereavement of loss of a spouse [30, 31]. These findings suggest continued engagement in life has a compensatory role for maintaining well-being and quality of life following adverse events during older adulthood [30]. Older adults residing in nursing homes also experience increases in quality of life from productive activities, such as perceiving oneself as being able to help others [25]. Furthermore, interpersonal relations (i.e., large social networks), as well as participation in activities, are suggested to increase the potential to age successfully by facilitating happiness, contentment, life satisfaction, and enjoyment [32, 33]. 

Increases in health have also been attributed to engagement in social, intellectual, cultural, leisure, and productive pursuits [29]. Individuals reporting higher levels of social engagement, irrespective of the activity, are significantly less likely to require health services and are prescribed less medication [34]. This finding is complemented by reports of increased objective and subjective health, reductions in all causes of mortality, and reductions in loneliness and depression through participation in social, productive, and physical activities [32, 35–37]. Taken together, these findings are highly suggestive that continued engagement in life provides an avenue to age successfully.

### 2.3. Engagement and the Built Environment

 The usual aging process is associated with natural changes in health and functioning [6]. These changes include (but are not limited to) reductions in muscle mass, the development of osteoporosis, and declines in cognitive and sensory function [6, 38–41]. Although these natural age-related changes have the potential to impact the ability to maintain an active engagement in life, the built environment can influence engagement profiles of older adults as well, both positively and negatively [19]. For example, the characteristics of the home environment can increase or reduce functional limitations of older adults by creating or removing barriers to daily tasks [42]. Furthermore, both residential and community designs that enable older adults to satisfy individual needs, and to access services and resources, are necessary to promote successful aging [22, 26]. Ultimately, these accommodating environments can increase well-being, quality of life, and participation in activity, all of which can aid in successful aging [25, 37]. In order to implement such environments to optimize the aging process, a comprehensive understanding of the theories of universal design is warranted.

## 3. Universal Design

### 3.1. More than Accessibility

The built environment has also been proposed to negatively impact older adults by creating barriers to maintain a continued engagement with life [18]. In consequence, social bonds and community connections can be severed due to an environment that hinders the functioning of this population [18]. However, environments that are thoughtfully designed or modified to accommodate varying abilities have the potential to reduce disability and foster engagement in social and productive activities in later life [43, 44]. Due to the impact the built environment has on daily functioning in older adulthood, it is imperative for designers to take into consideration changes experienced during the aging process [45]. 

Research specific to older adults and the built environment suggests that adults have a strong preference for aging in a place of familiarity, in regards to the home and broader community [18, 58]. Specifically, 75% of middle aged and older adults report a strong desire to reside as long as possible in their current homes [58]. Keeping in mind the current shift in demographics, such a preference creates a high demand for built environments that are accommodating and accessible to older adults. A number of design theories have principles that accommodate the aging process in the design of the built environment. In addition to universal design, these theories include accessible design, adaptable design, and transgenerational design (Table 2; [46, 48]). Although accessible and adaptable design features provide accommodations that could be helpful for older adults, these features are often fixed in place and noticeable [59] and may promote a sense of segregation among end-users [16] and of stigmatization as being “seniors” or “disabled” [60]. Since these features require specialized adaptations or equipment, they may also come at a substantial financial cost, particularly if they are added as retrofits to existing spaces [16]. Transgenerational design seeks to create products and environments that are compatible with the physical and sensory impairments associated with aging, often from the initial planning stages [48]. There is some overlap between universal design and transgenerational design: Farage et al. [61] maintain that many of the design principles of transgenerational design are consistent with the principles of universal design, and that thoughtful application of the universal design principles will lead to design that is transgenerational. However, since the focus of transgenerational design is on accommodating the adaptations that arise as a result of aging [62], accommodations for other populations may be overlooked [16], and this approach may still be stigmatizing due to its emphasis on “age” [62]. 

Though all thoughtful design has the potential to reduce or remove barriers associated with aging and disability; universal design strives to provide accessible environments without stigmatization or ageism, as the design principles are not specialized and are incorporated into the environment in the initial stages of planning [16, 18, 44]. Universal design is founded on the premise that there is only one population with varying characteristics rather than a “normal” and “diverging from normal” population [63]. “Because all groups are placed within the context of normal expectations of the human condition, trying to justify the importance of each vulnerable population group becomes unnecessary.” (Pastalan, as cited in [64], page 3). Thus, universal design removes segregation of the older population resulting in an ideal approach to increase successful aging throughout the lifespan. 

Universal design, often used interchangeably with inclusive design and design for all, is a 21st century professional, academic, and social movement [65, 66]. If implemented properly, universal design creates safe, accessible, and usable environments for the broadest spectrum of people possible [67]. Empirical evidence of the success of the universal design philosophy on the professional (i.e., architecture) and academic (i.e., theoretical application) fronts has been reported. Danford [68] provided a tour of a universally designed and a non-universally designed building to 24 adults with either a visual, auditory, or mobility impairment and eight adults with no impairments. All people, with and without impairments, reported less difficulty and required less effort and assistance to complete standard tasks (i.e., finding a public washroom, entering the building) in the building constructed using the universal design principles. 

The social movement of universal design has a theoretical foundation rather than an empirical framework. Currently, literature refers to universal design as a philosophy and a process, rather than a legal code and a result [63, 69]. While being mindful of this construct, universal design should be implemented as good practice as it not only incorporates building code requirements of design, it goes beyond the basic code requirements and provides equality regarding usability and accessibility among a wide spectrum of the population. Overall, this provides evidence that universal design is more than simply creating accessible environments; it encourages a shift in attitude toward democracy and equality for all citizens, including the continually growing older adult population [63]. With such an influence on society, it is plausible to propose universal design can facilitate continued engagement in life, and ultimately successful aging. 

### 3.2. A Step toward Successful Aging

The built environment can impact the engagement profiles of older adults both positively and negatively [18, 43, 44]. Therefore, it is important to consider specific examples of universal design that can allow for positive changes to engagement levels and ultimately promote successful aging. The recommendations provided in Table 3 are based on three basic activities of daily living (BADLs) and three instrumental activities of daily living (IADLs) that are identified in the literature as pertinent to the independence of older adults and are directly related to the built environment [70–72]. BADLs refer to activities of personal care and physical self-maintenance, whereas IADLs include community and domestic activities that require an individual to adapt to the environment [70, 71]. The implementation of the universal design options described in Table 3 would provide a stepping stone toward successful aging: Older adults would be provided with an opportunity to more easily accomplish BADLS and IADLs, which would promote independence in both social and productive activities and ultimately foster active engagement in life. 

### 3.3. Identifying and Overcoming Challenges

 Despite the positive implications universal design offers to adults experiencing natural age-related declines, this design philosophy is not without challenges and limitations. Primarily, the implementation of universal design is challenged by the minimal education provided to engineers, architects, and environmental planners regarding the importance and use of this design theory [73]. Furthermore, the lack of a standard education regarding universal design may partially account for the little consensus and consistency surrounding the definitions pertaining to universal design [63]. Secondly, universal design is limited in its implementation as we are currently at a time when norms and codes of practice take precedence over theory for guiding decisions and actions [63]. Universal design has a theoretical foundation and has yet to be adopted as “best practice” that goes beyond mandated codes and guidelines, and thus its principles are often overlooked. Thirdly, incorporating designs that simultaneously meet the needs of multiple end-users with widely diverse abilities into the built environment is challenging [62], and achieving this level of accommodation may not be realized if the architects assume an understanding of the barriers that the end-users will experience [74]. Despite the knowledge the architect may have regarding accessible design, and the sincerity of the architect's intentions to understand the end-users' specific needs; such needs are often difficult to interpret by able-bodied persons who may not experience them as barriers, and decisions regarding the application of accessible design within the built environment are often made with little or no input from end-users [75]. With respect to aging, architects may not be aware of the barriers presented to older adults within the built environment and may not recognize these individuals as those who may benefit from various accommodations. A fourth challenge regards the discrepancy between professionals and academics concerning the cost associated with implementing universal design. Academics have often reported that universal design can be implemented at little or no additional cost [16]. However, accessible and universal designs require more square footage within buildings and homes (i.e., larger bathroom stalls or residential bathrooms to allow wheelchair access and/or a caregiver), which will increase construction costs [65]. Finally, the architect's primary objective in designing any building is to meet the needs of the client (i.e., the decision makers). As such, an architect can provide suggestions to clients and rationalize them accordingly; however, an architect cannot force their values into a design. Therefore, the incorporation of universal design is dependent on the client's perceived importance of its implementation. Ultimately, if the client does not desire universal design and local building code does not require it, the architect will be unable to implement it. 

The aforementioned challenges to implementing universal design are obstacles that need to be overcome through a multidimensional approach. Future directions for professionals in architecture and environmental planning could include (1) increasing the incorporation of universal design into the educational curriculum while emphasizing the importance of applying the philosophy to the built environment; (2) communicating with older adults and persons with disabilities during the design process to ensure an understanding of the diversified needs of the population as a whole; (3) collaborating among one another to include universal design as a “best practice” guideline, making it a common implementation that exceeds minimal code requirements; and (4) develop more cost-effective ways to implement universal design in order to increase the affordability of such design, thereby increasing the client's willingness to include universal design principles in the design process [45]. The theory and literature surrounding universal design is very promising; however, many more randomized controlled trials testing the effectiveness of specific universal design options are needed. As a result of rigorous research and knowledge translation efforts, universally designed environments may become the norm and code of practice for architects and design professionals. As a society, the philosophy of universal design needs to be embraced with the aim of shifting individual attitudes towards perceiving the population as whole where individuals can equally thrive in the environment, regardless of ability or age. 

## 4. Conclusion

The concept of successful aging has become increasingly important as senior citizens begin to dominate the population demographics [3, 5]. Maintaining an active engagement in life through participation in social and productive pursuits is one component of successful aging [6, 7]. Since the built environment directly impacts the engagement profiles of older adults, it is necessary to provide environments designed to suit the needs of older adults [18]. Of all the design theories that attempt to accommodate the aging process, the philosophy of universal design may be the most desirable option as it provides built environments that benefit everyone, prevent stigmatization, and increase the ease of engagement in activities of daily living [16]. Currently, universal design is a promising voluntary philosophy that requires increased attention from facility planners and coordinators, evidence of the significance of its application from academics, and the embracement of its core principles by society. Taken as a whole, it is evident that the application of universal design to the built environment is the step toward successful aging that the graying population needs. 

## Figures and Tables

**Table 1:** Seven principles of universal design (adapted from Story [16]).

Universal design principle	Description	Example
(1) Equitable use	Useful and marketable to people with diverse abilities	Doors that automatically open

(2) Flexibility of use	Accommodates a wide range of individual preferences and abilities	Automated teller machines' buttons far enough apart to be pressed accurately

(3) Simple and intuitive use	Easy to understand, regardless of user's experience, knowledge, language skills, or current concentration level	Providing furniture assembly instructions in a series of clear illustrations instead of text

(4) Perceptible information	Communicates necessary information effectively to the user, regardless of ambient conditions or the user's sensory abilities	Computer software that relays information visually through text and pictures, and audibly through speakers

(5) Tolerance for error	Minimizes hazards and the adverse consequences of accidental or unintended actions	Hallways that return to common areas rather than stop in dead ends

(6) Low physical effort	Can be used efficiently and comfortably with a minimum of fatigue	Bottle caps that are easy to grip and require only a small range of motion to open

(7) Size/space for approach/use	Appropriate size and space is provided for approach, reach, manipulation, and use regardless of the user's body size, posture, or mobility	Wall mounted components (i.e., toilet paper) that are visible, easy to reach, and easy for all hand sizes to use

**Table 2:** Additional theories of design.

Design theory	Definition	Example
Accessible design [46]	Provides separate design features for user groups with disabilities Usually permanent and noticeable Fulfills code requirements for use by individuals with disabilities	Provide the minimum level of accessibility required by the local building code. This can vary by region (e.g., province or state), and with different building types within the same region. For example, Ontario Building Code requires power door operators (e.g., push button, automatic sensor, etc.) on entrances to hotels, but not on entrances to stand-alone office spaces of less than 300 m^2^ [47].

Adaptable design [46]	Provides design features that are usable by groups with disabilities, however remain concealed or omitted until needed Features are either adjustable or easily and quickly added or removed in order to “adapt” the environment for specific individuals	An electronic push button is provided to open the door, but the use of the push button is optional (i.e., door will open manually).

Transgenerational design [48]	Develops products and environments that are compatible with the natural physical and sensory declines experienced during the aging process	Provide a power assist door, which augments the force applied by the user to fully open the door [49].

**Table 3:** Examples of universal design techniques that will allow older adults to complete basic activities of daily living (BADLs) and instrumental activities of daily living (IADLs) with greater ease.

Activities of daily living	Examples of universal design
BADL	
Bathing	Make provisions during construction to reinforce walls in the shower area to facilitate future installation of grab bars [50] Bathtub/shower controls positioned to allow for operation outside the fixture [20] Lever handle faucets [20] No threshold walk-in shower [20]
Physical ambulation	No threshold, zero step entrances [50] Wider doorways and corridors [50] Open floor plan [20] Straight staircases with consistent risers and treads and a stopping place (landing) midway between levels [51]
Toileting	Make provisions during construction to reinforce bathroom walls to facilitate future installation of grab bars by the toilet [50] Installation of a downstairs bathroom [50] Adjustable toilet and sink for easy access, with a short reaching distance to paper dispenser and grab bars [20]

IADL	
Food preparation	Kitchen counter tops at varying levels to accommodate standing and seated users, and people of varying heights [52] Kitchen cabinets that accommodate limited reach ranges and allow various ways for approach and manipulation [53] Color contrasts, large-print readouts, audible and tactile feedback of controls [53] Close access to ovens with counter space directly next to the oven [53]
Shopping	Lowering or making height adjustable electronic devices used in typical purchasing transaction (i.e., credit card reader/swipe; [54]) Larger print on signs indicating aisle numbers and locations of goods, and on packaging of items [55] Larger aisle ways [55] Automatic powered doors at entrances and exits [56]
Transportation	Complement higher-order roads (i.e., interstates) with lower-speed, two lane through-routes [57] Connect local street networks within communities to create short drives and walking distances [57]

## References

[1] Anderson GF, Hussey PS (2000). Population aging: a comparison among industrialized countries. *Health Affairs*.

[2] Lloyd-Sherlock P (2000). Population ageing in developed and developing regions: implications for health policy. *Social Science and Medicine*.

[3] Statistics Canada (2012). The Canadian population in 2011: age and sex. *Catalogue*.

[4] Statistics Canada (2010). Population projections: Canada, the provinces and territories. *The Daily*.

[5] Lowry KA, Vallejo AN, Studenski SA (2012). Successful aging as a continuum of functional independence: lessons from physical disability models of aging. *Aging and Disease*.

[6] Rowe JW, Kahn RL (1987). Human aging: usual and successful. *Science*.

[7] Rowe JW, Kahn RL (1997). Successful aging. *The Gerontologist*.

[8] Tate RB, Lah L, Cuddy TE (2003). Definition of successful aging by elderly canadian males: the Manitoba Follow-Up Study. *The Gerontologist*.

[9] Del Bono E, Sala E, Hancock R, Gunnell C, Parisi L (2007). *Gender, Older People and Social Exclusion. A Gendered Review and Secondary Analysis of the Data*.

[10] Zunzunegui MV, Koné A, Johri M, Béland F, Wolfson C, Bergman H (2004). Social networks and self-rated health in two French-speaking Canadian community dwelling populations over 65. *Social Science and Medicine*.

[11] Levasseur M, Richard L, Gauvin L, Raymond E (2010). Inventory and analysis of definitions of social participation found in the aging literature: proposed taxonomy of social activities. *Social Science and Medicine*.

[12] Guralnik JM, Fried LP, Salive ME (1996). Disability as a public health outcome in the aging population. *Annual Review of Public Health*.

[13] Hertz JE, Anschutz CA (2002). Relationships among perceived enactment of autonomy, self-care, and holistic health in community-dwelling older adults. *Journal of Holistic Nursing*.

[14] Thorpe KE, Philyaw M (2012). The medicalization of chronic disease and costs. *Annual Review of Public Health*.

[15] Lawton MP (1983). Environment and other determinants of well-being in older people. *The Gerontologist*.

[16] Story MF (1998). Maximizing usability: the principles of universal design. *Assistive Technology*.

[17] Ontario Ministry of Community and Social Services (2008). About the Accessibility for Ontarians with Disabilities Act (AODA), 2005. *Catalogue*.

[18] Demirkan H (2007). Housing for the aging population. *European Review of Aging and Physical Activity*.

[19] Crews DE, Zavotka S (2006). Aging, disability, and frailty: implications for universal design. *Journal of Physiological Anthropology*.

[20] DeMerchant EA, Beamish JO (1995). Universal design in residential spaces. *Housing and Society*.

[21] Mendes de Leon CF (2005). Social engagement and successful aging. *European Journal of Ageing*.

[22] Bowling A, Dieppe P (2005). What is successful ageing and who should define it?. *British Medical Journal*.

[23] Charbonneau-Lyons DL, Mosher-Ashley PM, Stanford-Pollock M (2002). Opinions of college students and independent-living adults regarding successful aging. *Educational Gerontology*.

[24] Phelan EA, Anderson LA, LaCroix AZ, Larson EB (2004). Older adults’ views of “successful aging”—how do they compare with researchers’ definitions?. *Journal of the American Geriatrics Society*.

[25] Guse LW, Masesar MA (1999). Quality of life and successful aging in long-term care: perceptions of residents. *Issues in Mental Health Nursing*.

[26] Fisher BJ, Specht DK (1999). Successful aging and creativity in later life. *Journal of Aging Studies*.

[27] Knight T, Ricciardelli LA (2003). Successful aging: perceptions of adults aged between 70 and 101 years. *International Journal of Aging and Human Development*.

[28] A J, Liffiton, Horton S, Baker J, Weir PL (2012). Successful aging: how does physical activity influence engagement with life?. *European Review of Aging and Physical Activity*.

[29] Fone S, Lundgren-Lindquist B (2003). Health status and functional capacity in a group of successfully ageing 65–85 years olds. *Disability and Rehabilitation*.

[30] Silverstein M, Parker MG (2002). Leisure activities and quality of life among the oldest old in Sweden. *Research on Aging*.

[31] Bergstrom MJ, Holmes ME (2000). Lay theories of successful aging after the death of a spouse: a network text analysis of bereavement advice. *Health Communication*.

[32] Brown CA, McGuire FA, Voelkl J (2008). The link between successful aging and serious leisure. *International Journal of Aging and Human Development*.

[33] Strawbridge WJ, Wallhagen MI, Cohen RD (2002). Successful aging and well-being: self-rated compared with Rowe and Kahn. *The Gerontologist*.

[34] Bath PA, Gardiner A (2005). Social engagement and health and social care use and medication use among older people. *European Journal of Ageing*.

[35] Bennett KM (2005). Social engagement as a longitudinal predictor of objective and subjective health. *European Journal of Ageing*.

[36] Glass TA, Mendes de Leon C, Marottoli RA, Berkman LF (1999). Population based study of social and productive activities as predictors of survival among elderly Americans. *British Medical Journal*.

[37] Grundy E, Bowling A (1999). Enhancing the quality of extended life years. Identification of the oldest old with a very good and very poor quality of life. *Aging and Mental Health*.

[38] Hedden T, Gabrieli JDE (2004). Insights into the ageing mind: a view from cognitive neuroscience. *Nature Reviews Neuroscience*.

[39] Lang T, Streeper T, Cawthon P, Baldwin K, Taaffe DR, Harris TB (2010). Sarcopenia: etiology, clinical consequences, intervention, and assessment. *Osteoporosis International*.

[40] Lindenberger U, Ghisletta P (2009). Cognitive and sensory declines in old age: gauging the evidence for a common cause. *Psychology and Aging*.

[41] Park DC, Reuter-Lorenz P (2009). The adaptive brain: aging and neurocognitive scaffolding. *Annual Review of Psychology*.

[42] Wahl HW, Fänge A, Oswald F, Gitlin LN, Iwarsson S (2009). The home environment and disability-related outcomes in aging individuals: what is the empirical evidence?. *The Gerontologist*.

[43] Liu SY, Lapane KL (2009). Residential modifications and decline in physical function among community-dwelling older adults. *The Gerontologist*.

[44] Verbrugge LM, Jette AM (1994). The disablement process. *Social Science and Medicine*.

[45] Demirbilek O, Demirkan H (2004). Universal product design involving elderly users: a participatory design model. *Applied Ergonomics*.

[46] Centre for Universal Design Definitions: Accessible, Adaptable, and Universal Design. http://www.ncsu.edu/www/ncsu/design/sod5/cud/pubs_p/phousing.htm.

[47] Building Code Act Ontario Regulation 350/06, Sentence 3. 8. 3. 3.(4). http://www.search.e-laws.gov.on.ca/en/isysquery/cbd0d22c-40df-4754-b58e-e261ddd42ecb/4/doc/?search=browseStatutes&context=#hit1.

[48] Pirkl JJ (1995). Transgenerational design: prolonging the American dream. *Generations*.

[49] Steinfeld E, Danford GS Automated Doors: Towards Universal Design. http://www.ap.buffalo.edu/idea/publications/automateddoors.htm.

[50] Weisman LK (2000). Creating the universal designed city: prospects for the new century. *Architectural Theory Review*.

[51] Leibrock C, Behar S (1993). *Beautiful Barrier-Free: A Visual Guide to Accessibility*.

[52] Marsden JP, Meehan RA, Calkins MP (2001). Therapeutic kitchens for residents with dementia. *American Journal of Alzheimer’s Disease and other Dementias*.

[53] Afacan Y, Demirkan H (2010). A priority-based approach for satisfying the diverse users’ needs, capabilities and expectations: a universal kitchen design case. *Journal of Engineering Design*.

[54] Bajaj K, Mirka GA, Sommerich CM, Khachatoorian H (2006). Evaluation of a redesigned self-checkout station for wheelchair users. *Assistive Technology*.

[55] Kaufman CF (1995). Shop ’til you drop: tales from a physically challenged shopper. *Journal of Consumer Marketing*.

[56] Story MF, Mueller JL, Mace RL The universal design file: designing for people of all ages and abilities. http://design-dev.ncsu.edu/openjournal/index.php/redlab/article/view/102.

[57] Dumbaugh E (2008). Designing communities to enhance the safety and mobility of older adults: a universal approach. *Journal of Planning Literature*.

[58] American Association of Retired Persons (2010). *Home and Community Preferences of the 45+ Population*.

[59] Deardorff CJ, Birdsong C (2003). Universal design: clarifying a common vocabulary. *Housing and Society*.

[60] Audirac I (2008). Accessing transit as universal design. *Journal of Planning Literature*.

[61] Farage MA, Miller KW, Ajayi F, Hutchins D (2012). Design principles to accommodate older adults. *Global Journal of Health Science*.

[62] Keates S, Clarkson PJ, Harrison LA, Robinson P Towards a practical inclusive design approach.

[63] Iwarsson S, Ståhl A (2003). Accessibility, usability and universal design—positioning and definition of concepts describing person-environment relationships. *Disability and Rehabilitation*.

[64] Center for Universal Design Universal design. Housing for the lifespan of all people. http://design-dev.ncsu.edu/openjournal/index.php/redlab/article/view/101.

[65] Commission for Architecture and the Built Environment (2008). *Inclusion By Design: Equality, Diversity and the Built Environment*.

[66] Froyen H, Verdonck E, De Meester D, Heylighen A (2009). Documenting handicap situations and eliminations through universal design patterns. *Australasian Medical Journal*.

[67] Kose S (1998). From barrier-free to universal design: an international perspective. *Assistive Technology*.

[68] Danford GS (2003). Universal design people with vision, hearing, and mobility impairments evaluate a model building. *Generations*.

[69] Trost G (2005). State of affairs in universal design in Europe. *Fujitsu Scientific and Technical Journal*.

[70] Fricke J Activities of Daily Living. http://hdl.handle.net/1959.9/160364.

[71] Katz S (1983). Assessing self-maintenance: activities of daily living, mobility, and instrumental activities of daily living. *Journal of the American Geriatrics Society*.

[72] Lawton MP, Brody EM (1969). Assessment of older people: self-maintaining and instrumental activities of daily living. *The Gerontologist*.

[73] Aslaksen F, Bergh S, Bringa OR, Heggem EK (1997). *Universal Design: Planning and Design for All*.

[74] Hall P, Imrie R (1999). Architectural practices and disabling design in the built environment. *Environment and Planning B*.

[75] Luck R, Haenlein H, Bright K (2001). Project briefing for accessible design. *Design Studies*.

